# Modulation of microRNA Expression in Subjects with Metabolic Syndrome and Decrease of Cholesterol Efflux from Macrophages via microRNA-33-Mediated Attenuation of ATP-Binding Cassette Transporter A1 Expression by Statins

**DOI:** 10.1371/journal.pone.0154672

**Published:** 2016-05-03

**Authors:** Wei-Ming Chen, Wayne H-H Sheu, Pei-Chi Tseng, Tzong-Shyuan Lee, Wen-Jane Lee, Pey-Jium Chang, An-Na Chiang

**Affiliations:** 1 Institute of Biochemistry and Molecular Biology, National Yang-Ming University, Taipei, Taiwan; 2 Division of Gastroenterology and Hepatology, Department of Internal Medicine, Chang Gung Memorial Hospital, Chiayi, Taiwan; 3 Graduate Institute of Clinical Medical Sciences, College of Medicine, Chang Gung University, Taoyuan, Taiwan; 4 Division of Endocrinology and Metabolism, Department of Internal Medicine, Taichung Veterans General Hospital, Taichung, Taiwan; 5 School of Medicine, National Yang-Ming University, Taipei, Taiwan; 6 School of Medicine, National Defense Medical Center, Taipei, Taiwan; 7 Institute of Medical Technology, National Chung-Hsing University, Taichung, Taiwan; 8 Institute of Physiology, National Yang-Ming University, Taipei, Taiwan; University of Catanzaro, ITALY

## Abstract

Metabolic syndrome (MetS) is a complicated health problem that encompasses a variety of metabolic disorders. In this study, we analyzed the relationship between the major biochemical parameters associated with MetS and circulating levels of microRNA (miR)-33, miR-103, and miR-155. We found that miRNA-33 levels were positively correlated with levels of fasting blood glucose, glycosylated hemoglobin A1c, total cholesterol, LDL-cholesterol, and triacylglycerol, but negatively correlated with HDL-cholesterol levels. In the cellular study, miR-33 levels were increased in macrophages treated with high glucose and cholesterol-lowering drugs atorvastatin and pitavastatin. miR-33 has been reported to play an essential role in cholesterol homeostasis through ATP-binding cassette transporter A1 (ABCA1) regulation and reverse cholesterol transport. However, the molecular mechanism underlying the linkage between miR-33 and statin treatment remains unclear. In the present study, we investigated whether atorvastatin and pitavastatin exert their functions through the modulation of miR-33 and ABCA1-mediated cholesterol efflux from macrophages. The results showed that treatment of the statins up-regulated miR-33 expression, but down-regulated *ABCA1* mRNA levels in RAW264.7 cells and bone marrow-derived macrophages. Statin-mediated *ABCA1* regulation occurs at the post-transcriptional level through targeting of the 3′-UTR of the *ABCA1* transcript by miR-33. Additionally, we found significant down-regulation of ABCA1 protein expression in macrophages treated with statins. Finally, we showed that high glucose and statin treatment significantly suppressed cholesterol efflux from macrophages. These findings have highlighted the complexity of statins, which may exert detrimental effects on metabolic abnormalities through regulation of miR-33 target genes.

## Introduction

MicroRNAs (miRs; miRNAs) are small (approximately 22 nucleotides in length) single-stranded noncoding RNAs that usually regulate target gene expression by post-transcriptional regulation [1,2]. Binding to the 3′-UTR is the major mechanism by which miRNAs promote the degradation of target messenger RNA (mRNA) molecules and inhibit protein expression [3,4]. It has been reported that miRNAs can be transported between cells and tissues via the circulatory system and can act as regulators of many metabolic processes [5]. Furthermore, miRNAs are strongly associated with systemic diseases, including metabolic syndrome, diabetes, and cardiovascular disease [6–9]. In particular, miR-33, miR-103, and miR-155 have revealed the possible functions related to cholesterol transportation, insulin resistance, and glucose homeostasis, respectively [10–12]. We thus hypothesize that expression levels of miR-33, miR-103, and miR-155 may be correlated with metabolic abnormalities and thus may potentially serve as the therapeutic target(s) for metabolic diseases.

Metabolic syndrome (MetS) is characterized by increased waist circumference, hyperglycemia, hypertriglyceridemia, low high-density lipoprotein (HDL)-cholesterol, and hypertension [13]. Subjects with MetS are at high risk for the development of type 2 diabetes and cardiovascular diseases. Accumulation of cholesterol in arterial macrophages has a major impact on the progression of atherosclerotic cardiovascular disease, whereas ATP-binding cassette transporter A1 (ABCA1) plays an important role in exporting excess cellular cholesterol to HDL through apolipoprotein A1 (apoA1) and reduces cholesterol accumulation in macrophages [14]. Consequently, ABCA1 is crucial for reverse cholesterol transport (RCT), the process of removing cholesterol from peripheral tissues back to the liver for excretion. Growing evidence suggests that impaired expression of ABCA1 or abnormal HDL function increases the risk for MetS, type 2 diabetes, and atherosclerotic progression [15–18]. Therefore, ABCA1 appears to be a promising therapeutic target for metabolic syndrome. ABCA1 expression has been reported to be regulated by transcriptional and posttranscriptional processes [19,20]. Previously, we have shown that hyperglycemia suppresses ABCA1 expression via post-transcriptional regulation in macrophages [21]. In this study, we investigated the molecular mechanisms underlying statin-modulated ABCA1 expression and cholesterol efflux from macrophages, which may have therapeutic intervention for the treatment of cardiometabolic disorders.

Statins are 3-hydroxy-3-methylglutaryl coenzyme A (HMG-CoA) reductase inhibitors with pleiotropic effects independent of cholesterol biosynthesis, including endothelial function improvement, anti-oxidative effects, and anti-inflammatory effects [22–24]. Statins are usually used in reducing serum cholesterol levels and the incidence of cardiovascular events. Atorvastatin and pitavastatin are potent and widely used lipophilic statins. It has been shown that lipophilic statins easily diffuse into the intracellular space and also decrease insulin secretion in response to glucose challenge [25]. The effectiveness of atorvastatin in lowering low density lipoprotein-cholesterol (LDL-C) levels has been demonstrated in several trials [26,27]. Furthermore, atorvastatin treatment has also been found to decrease phosphorylation of IRS-1 and Akt [28], down-regulate expression of glucose transporter 4 (GLUT4), and reduce glucose uptake [29] in adipocytes. In contrast, pitavastatin is a statin that significantly increases HDL-C in patients with MetS and type 2 diabetes mellitus [30,31]. Although statins confer a substantial reduction of cardiovascular risk [32], emerging evidence suggests that treatment with statins may increase the risk of hyperglycemia and new-onset diabetes [33–35]. This significant residual risk in statin-treated diabetes still remains an unsolved problem for physicians [36–38]. In 2012, the US Food and Drug Administration (FDA) provided evidence supporting the use of warning labels regarding the risk of statins [39]. It is essential to elucidate the mechanisms underlying the potential unforeseen effects of statins use in clinical intervention.

Dysfunctional cholesterol homeostasis is linked to MetS, type 2 diabetes, and atherosclerosis. Some miRNAs regulate cholesterol homeostasis and are possibly used as the therapeutic targets in cardiovascular medicine [9,10,40,41]. Several reports show that miR-33 contributes to regulation of cholesterol homeostasis, which may promote the progress of atherosclerosis [42,43]. This study demonstrates that silencing of miR-33 in mice increases circulating HDL levels and promotes RCT due to increased ABCA1-dependent cholesterol efflux. However, the impact of statins on regulation of miRNA expression and its contribution to cholesterol metabolism in macrophages has not been reported previously. In the present study, we discovered a statin-miR-33-ABCA1 regulatory pathway that modulates cholesterol homeostasis in macrophages.

## Materials and Methods

### Study design

This study was designed as a prospective study and the study protocol was approved by the ethics committee of the Chang Gung Medical Foundation Institutional Review Board. All subjects (65 healthy controls and 74 MetS patients) gave their written informed consent before participating in the study. The investigators were blinded to each subject’s group allocation and treatment. None of the subjects had previous exposure to statins. Subjects were excluded from the study if they had signs of acute infection, renal failure (serum creatinine levels > 3 mg/dL), mental illness, and/or severe systemic diseases. The physical examinations included anthropometric measurements and blood tests. Body mass index (BMI) was calculated as weight (kg)/height (m)^2^. MetS was defined according to the presence of any 3 of the following 5 criteria: waist circumference ≥90 cm in men and ≥80 cm in women; fasting plasma glucose ≥ 100 mg/dL or glycosylated hemoglobin (HbA1c) ≥ 6.5%; HDL cholesterol ≤ 40 mg/dL in men and 50 mg/dL in women; hypertriglyceridemia ≥ 150 mg/dL; and systolic blood pressure (BP) ≥130 mmHg or diastolic BP ≥ 85 mmHg. The baseline characteristics of the study subjects are shown in Table 1. Subjects with MetS were randomly assigned to groups that received atorvastatin (10 mg/day) or pitavastatin (1 mg/day) treatment. There were no significant differences in age and BMI between the atorvastatin and pitavastatin groups. Peripheral blood was collected from subjects after an overnight fast and plasma was separated and stored at -70°C until the analysis.

**Table 1:** Clinical characteristics of the study subjects.

	Health controls (n = 65)	Metabolic syndrome cases (n = 74)	*P*
Gender (M/F)	34/31	39/35	
Age (years)	57.4 ± 6.4	57.7 ± 5.5	n.s.
BMI (kg/m2)	23.8 ± 1.9	29.4 ± 2.6	<0.001
Systolic BP (mmHg)	126 ± 12.3	151 ± 9.0	<0.001
Diastolic BP (mmHg)	72 ± 9.6	94 ± 8.8	<0.001
FBG (mg/dL)	92.4 ± 13.4	121.7 ± 26.3	<0.001
HbA1c (%)	6.0 ± 0.9	8.2 ± 1.2	<0.001
TC (mg/dL)	189 ± 22.7	224 ± 26.7	<0.001
HDL-C (mg/dL)	52.4 ± 7.4	40.6 ± 7.6	<0.001
LDL-C (mg/dL)	112 ± 22.6	151 ± 27.4	<0.001
TG (mg/dL)	111 ± 24.1	122 ± 36.3	<0.05

### Laboratory analyses

Fasting blood glucose (FBG) and plasma levels of total cholesterol (TC), high-density lipoprotein cholesterol (HDL-C), low-density lipoprotein cholesterol (LDL-C), and triacylglycerol (TG) were measured using an automatic analyzer (Hitachi 7600 Modular, Japan). Hemoglobin (Hb) A1C was measured with a high performance liquid chromatography (HPLC) system (Trinity Biotech Premier Hb9210 analyzer, Kansas City, MO, USA). Total RNA was isolated from human plasma using Trizol reagent (Invitrogen, Carlsbad, CA, USA). microRNA was prepared using the mirVANA microRNA isolation kit (Ambion, Austin, TX, USA) according to the manufacturer’s instructions. RNA concentrations were determined using a Nanodrop 1000 spectrophotometer (Nanodrop Technologies). A total of 2 μg RNA was used for producing cDNA using the TaqMan microRNA reverse transcription kit (Applied Biosystems, Foster City, CA, USA). The real-time PCR reactions for the plasma miRNA assays were performed in a 20-μL final volume containing 2 μL of cDNA using the TaqMan universal PCR Master Mix and TaqMan microRNA assay primers for human miR-33a, miR-103, and miR-155 (Applied Biosystems). Expression levels of cel-miR-39, RNU6B, and mir-16 were used as the endogenous controls for assays of miR-33, miR-103, and miR-155, respectively. To quantify the relative expression level of each miRNA, threshold cycle (Ct) values were normalized against endogenous reference (ΔCt = Ct (target miR)−Ct (endogenous control)). Expression levels of human plasma miRNAs were calculated by the 2^−ΔCt^ method. To determine the absolute copy number of miRNAs, standard curves was prepared for each miRNA by serial dilution of synthetic miRNAs. The Ct values for each sample reaction were converted to absolute copy number based on its standard curve. The values of miRNA copy number were quantified per one ng of total RNA.

### Cell culture and miR-33 assessments

Murine macrophage cell line RAW264.7 was originally obtained from the American Type Culture Collection (Manassas, VA, USA) and grown in Dulbecco’s modified Eagle’s medium (DMEM; Hyclone Laboratories, Logan, UT, USA) supplemented with 10% fetal bovine serum (FBS), 100 units/mL penicillin, and 100 μg/mL streptomycin. All cell experiments were performed in a humidified atmosphere at 37°C with 5% CO_2_. Bone marrow-derived macrophages (BMDMs) were generated as previously described [44]. Briefly, bone marrow cells were isolated from mouse femurs and cultured in Petri dishes with DMEM containing 20% FBS, 100 units/mL penicillin, 100 μg/mL streptomycin, and 50 ng/mL macrophage colony-stimulating factor (M-CSF; R & D Systems, Minneapolis, MN, USA) for 7 days. Bone marrow cells were collected by centrifugation at 2000 rpm for 5 min and cultured in DMEM supplemented with 10% FBS, 100 units/mL penicillin, 100 μg/mL streptomycin, and 50 ng/mL M-CSF. BMDM cells were plated into culture dishes in the presence of 20% L929 media and left to differentiate for 7–8 days. Cells were lifted into suspension in the existing L929-supplemented DMEM by gentle scraping and seeded into the appropriate plate for subsequent experiments. Trizol reagent (Invitrogen, Carlsbad, CA, USA) was used to extract total RNA from cellular lysates and medium from macrophages treated with glucose (5 to 30 mM) or statin (1 to 10 μM) for 24 h. Preparation of microRNA was performed using the mirVANA microRNA isolation kit (Ambion, Austin, TX, USA) according to the manufacturer’s instructions. Relative miR-33 expression was calculated via the 2^−ΔΔCt^ method with the TaqMan microRNA reverse transcription kit and fold changes were calculated. cel-miR-39 was used as an endogenous control. To improve the accuracy of real-time RT-PCR for miR-33 quantification, amplifications were performed at least in triplicate for each RNA sample.

### Reporter plasmids, transient transfections, and luciferase assays

The *ABCA1* promoter-driven luciferase reporter plasmid was constructed using the mouse *ABCA1* promoter region spanning from -250 to -1 bp, which shows strong promoter activity in macrophages [45]. Primers for the *ABCA1* promoter were the following: forward, 5′-TGCCTCGAGGGCCAGGGCTACAGAAAGCGG-3′ and reverse, 5′-CCGAAGCTTGGTTTTTGCCGCGACTAGTTC-3′. The underlined sequences represent *Xho*I and *Hin*dIII sites, respectively. The PCR-amplified DNA fragment containing the *ABCA1* promoter was cloned into *Xho*I and *Hin*dIII sites of the luciferase reporter plasmid pGL3-basic vector (Promega, Madison, WI, USA). The 3′-UTR of the *ABCA1* mRNA contains three highly conserved consensus-binding sites for miR-33a [10]. The cDNA fragment corresponding to 20 to 210 bp downstream of the murine *ABCA1* mRNA was amplified by PCR from total RNA template extracted from RAW264.7 macrophages. The sequences of primers used for PCR amplification were as follows: forward, 5′-TGATCTAGAGTGAATGAAAGGAAGGAAGAGCGAGG-3′ and reverse, 5′-GCGTCTAGAATACAAGACATAGGCTACAAAGGCAC-3′. The underlined sequences represent the *Xba*I site. This PCR product was digested with *Xba*I and was ligated into the *Xba*I site (downstream of the luciferase gene) of the *ABCA1* promoter-driven luciferase reporter plasmid to generate the pGL3-*ABCA1* plasmid. The DNA sequence of the construct was verified by automated DNA sequencing. Approximately 2.5 × 10^5^ RAW264.7 macrophages were seeded per well in 6-well plates in the growth medium DMEM, which contains 10% FBS, 1% L-glutamine, 1% nonessential amino acids, 1% sodium pyruvate, and 1% penicillin-streptomycin. The next day, medium was changed to 300 μl serum free DMEM per well and cells were transiently co-transfected with 1 μg of the pGL3-*ABCA1* and 0.5 μg of the pCMV-β-galactosidase expression plasmid using Lipofectamine LTX and Lipofectamine PLUS reagents (Invitrogen) according to the manufacturer’s protocol. After treatment with atorvastatin and pitavastatin from 1 to 10 μM for 24 h, cells were lysed in 50 μl lysis buffer (Promega, Madison, WI, USA) for 15 min and luciferase activity was determined using a VICTOR^2^ Multilabel Reader (PerkinElmer). Luciferase activity was normalized to β-galactosidase activity and expressed as ratios relative to the values obtained from the untreated pGL3-*ABCA1* control group defined as 1.0.

### Analysis of *ABCA1* gene expression

Total cellular RNA was extracted using the Trizol reagent following the manufacturer’s instructions. Reverse transcriptase (RT)-PCR was performed by SuperScript^™^ III First-Strand Synthesis System for RT-PCR (Invitrogen, Life Technologies). Specific primers were used for the PCR amplifications of *ABCA1* (forward, 5′-CAATGCCCCTCTTCATGACT-3′; reverse, 5′-TGCAGTGGTGAGATTGAAGC-3′) and actin (forward, 5′-CCAGAGCAAGAGAGGTAT-3′; reverse, 5′-ATAGAGGTCTTTACGGATGT-3′). PCR products were separated on 2% agarose gels and visualized by ethidium bromide staining. The level of *ABCA1* gene expression was normalized using actin as the internal control.

Regulation of *ABCA1* mRNA was also quantified by real-time RT-PCR using specific primers for *ABCA1* (forward, 5′-GGTTTGGAGATGGTTATACAATAGTTGT-3′; reverse, 5′-CCCGGAAACGCAAGTCC-3′) and glyceraldehyde 3-phosphate dehydrogenase (GAPDH; forward, 5′-GTATGACTCCACTCACGGCAAA-3′; reverse, 5′-GGTCTCGCTCCTGGAAGATG-3′). The reaction conditions were as follows: 10 min at 95°C followed by 40 cycles of 20 s at 95°C, 20 s at 55°C, and 20 s at 72°C. Relative mRNA expression levels were normalized to that of porphobilinogen deaminase (*PBGD*) (ΔCt = Ct (*ABCA1*)−Ct (*PBGD*)) in each assay. Quantification of *ABCA1* mRNA was performed using the 2^−ΔΔCt^ method.

### Western blotting analysis

Cells were lysed with lysis buffer [1 mM EDTA, 1 mM EGTA, 1% Triton X-100, 20 mM Tris-HCl, pH 7.5, phosphatase inhibitor cocktail I and II (Sigma-Aldrich), 50 mM dithiothreitol, 150 mM NaCl, and complete protease inhibitor cocktail (Roche Diagnostics)], after which cell lysates were resuspended in 5×Tris-glycine SDS sample buffer. The protein concentration was assayed using Bradford reagent (Bio-Rad, Hercules, CA, USA). Equal amounts of protein from each sample were subjected to SDS polyacrylamide gel electrophoresis and transferred to nitrocellulose membranes (Pall, Glen Cove, NY, USA). The immunoblots were blocked with 5% non-fat milk in PBST (5.8 mM Na_2_HPO_4_·7H_2_O, 130 mM NaCl, and 0.05% Tween-20) at room temperature and incubated with anti-ABCA1 antibodies (Abcam, Cambridge, UK) overnight at 4°C. After the blots were washed with PBST, they were incubated with horseradish peroxidase (HRP)-conjugated secondary antibodies (Sigma-Aldrich), and target protein bands were detected using an enhanced chemiluminescence system (ECL; PerkinElmer). Next, the blots were stripped to allow further probing with anti-α-tubulin antibodies as an internal control for the amount of total cellular protein. The relative intensities of the protein bands were quantified by densitometry using ImageQuant software (Molecular Dynamics, Sunnyvale, CA, USA).

### Cholesterol efflux assessments

RAW264.7 macrophages and BMDM cells were plated separately with 0.5 μCi/mL [1,2-^3^H]cholesterol (radiolabeled cholesterol) (PerkinElmer, Boston, MA) in DMEM containing 0.2% BSA for 24 h. Cells were also treated with cholesterol (Wako Pure Chemical Industries, Osaka, Japan) dissolved in ethanol and diluted in serum-free medium. RAW264.7 macrophages were incubated in DMEM with 10 μg/mL of acetylated low-density lipoprotein (AcLDL; Molecular Probes, Eugene, OR, USA) and 30 μg/mL of cholesterol in the presence of 0.5 μCi/mL [1,2-^3^H]cholesterol for 24 h. Subsequently, cells were washed and incubated with glucose (5 to 30 mM) or statins (1 and10 μM atorvastatin or pitavastatin) for another 24 h. To perform apoAI-mediated cholesterol efflux, the medium was replaced with fresh medium containing 0.2% BSA with or without 10 μg/mL lipid-free human apoAI (Sigma-Aldrich) for 24 h. The efflux medium was collected and centrifuged to remove cell debris. Cells were lysed in 0.1N NaOH and radioactivity was measured by liquid scintillation counting. The cholesterol efflux to apoAI was calculated by subtracting the nonspecific efflux of cells exposed to BSA without apoAI. Cholesterol efflux was expressed as the percentage of the counts measured from the medium relative to the total counts measured from the medium and cell lysate.

### Statistical analyses

Data are presented as mean ± SEM. All results are representative of three to five separate experiments. Differences between two groups were compared using two-tailed Student’s *t*-test. Spearman’s correlation coefficients were used to examine the relationship between each biochemical parameter and specific miRNA levels. The non-parametric Wilcoxon signed-rank test was performed to assess changes in human plasma miRNA levels after statin treatment. For the cellular studies, intergroup comparisons of means were performed by ANOVA followed by Tukey’s test. *P*-values less than 0.05 were considered to be statistically significant.

## Results

### Correlation between miRNA expression and biochemical parameters

The study included a total of 74 subjects (39 men and 35 women) with metabolic syndrome and 65 healthy controls (34 men and 31 women). The plasma levels of relative miR-33, miR-103, and miR-155 expression were 2.1±0.3, 4.4±0.9, 5.5±1.0, respectively, in the healthy subjects. Significantly higher levels of miR-33 (22.3±6.1; *P*<0.01) and miR-103 (10.2±2.10; *P*<0.05) were observed in the MetS group, whereas the levels of miR-155 expression (3.2±0.7) were not significantly changed in the MetS group. Compared to the mean values of miR-33, miR-103, and miR-155 copy number per ng of total RNA in healthy controls with the 33±5, 282±53, and 112±21 copies, the mean copy numbers of miR-33 (206±54; *P*<0.01) and miR-103 (711±153; *P*<0.05) were also significantly higher in the MetS group, whereas the copy numbers of miR-155 (150±19) were not significantly changed. When we compared the correlations between biochemical parameters and miRNA levels in all subjects (Table 2), miR-33 levels were found to be positively correlated with fasting blood glucose (r = 0.594, *P* < 0.001), HbA1c (r = 0.384, *P* < 0.001), total cholesterol (r = 0.202, *P* < 0.05), LDL-cholesterol (r = 0.33, *P* < 0.001), and triacylglycerol levels (r = 0.22, *P* < 0.01), but negatively correlated with HDL-C levels (r = −0.689, *P* < 0.001). Plasma levels of miR-103 were also positively correlated with fasting blood glucose (r = 0.25, *P* < 0.01) and triacylglycerol levels (r = 0.178, *P* < 0.05), but negatively correlated with HDL-C levels (r = −0.327, *P* < 0.001). However, miR-155 levels were not significantly correlated with the measured biochemical parameters.

**Table 2:** Correlation between biochemical parameters and miRNA levels in all subjects.

	miR-33		miR-103		miR-155	
	r	*P*	r	*P*	r	*P*
FBG (mg/dL)	0.594	<0.001	0.250	<0.01	−0.010	n.s.
HbA1c (%)	0.384	<0.001	0.102	n.s.	−0.154	n.s.
TC (mg/dL)	0.202	<0.05	0.041	n.s.	−0.014	n.s.
HDL-C (mg/dL)	−0.689	<0.001	−0.327	<0.001	−0.060	n.s.
LDL-C (mg/dL)	0.330	<0.001	0.109	n.s.	0.026	n.s.
TG (mg/dL)	0.220	<0.01	0.178	<0.05	0.164	n.s.

### Activation of miRNA-33 expression by glucose and statin treatment

**Fig 1:**
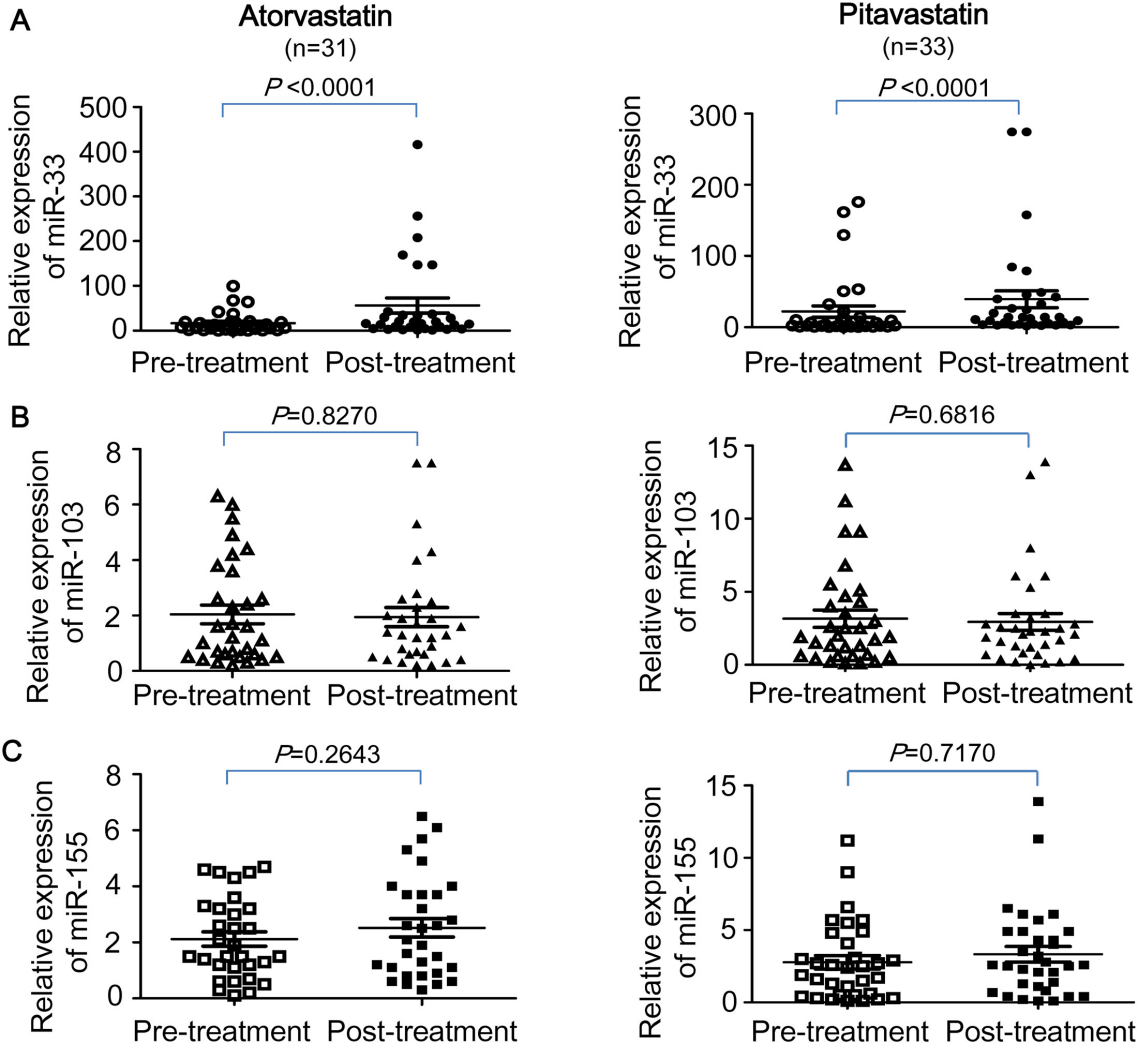
Relative plasma levels of miR-33, miR-103, and miR-155 in subjects before and after treatment with atorvastatin and pitavastatin. Total RNA was isolated from human plasma and RNA concentrations were determined using a Nanodrop 1000 spectrophotometer. cDNA was generated from the RNA and microRNA assays were performed by real-time PCR using the specific primers for human miR-33a, miR-103, and miR-155. Expression levels of cel-miR-39, RNU6B, and mir-16 were used as the endogenous controls for assays of miR-33, miR-103, and miR-155, respectively. To quantify the relative expression level of each miRNA, threshold cycle (Ct) values were normalized against endogenous reference (ΔCt = Ct (target miR)−Ct (endogenous control)). Expression levels of human plasma miRNAs were calculated by the 2^−ΔCt^ method. Each microRNA was assayed in triplicates.

A total of 64 subjects completed the 3-month statin therapy, in which 31 cases were treated with atorvastatin and 33 cases were treated with pitavastatin. As shown in Fig 1, significant increases in plasma miR-33 levels were found in subjects treated with atorvastatin or pitavastatin (*P*<0.0001), while no significant change in the relative expression of plasma miR-103 or miR-155 was shown after statin treatment. Compared to the relative expression levels of miR-33 before atorvastatin (17±4) and pitavastatin (22±8) treatment, the miR-33 levels were increased by 3.3–fold and 1.8–fold, respectively, in MetS patients after treatment with atorvastatin (56±17) and pitavastatin (39±12). The absolute levels of plasma miR-33 copy number (per ng total RNA) were also significantly higher in MetS patients after treatment with atorvastatin (512±114 copies; *P*<0.0001) and pitavastatin (637±239 copies; *P*<0.0001) compared to themselves before treatment of atorvastatin (162±33 copies) and pitavastatin (226±104 copies). However, no significant change in both relative expression levels and absolute copy numbers of miR-103 and miR-155 was found in MetS patients treated with either statin.

**Fig 2:**
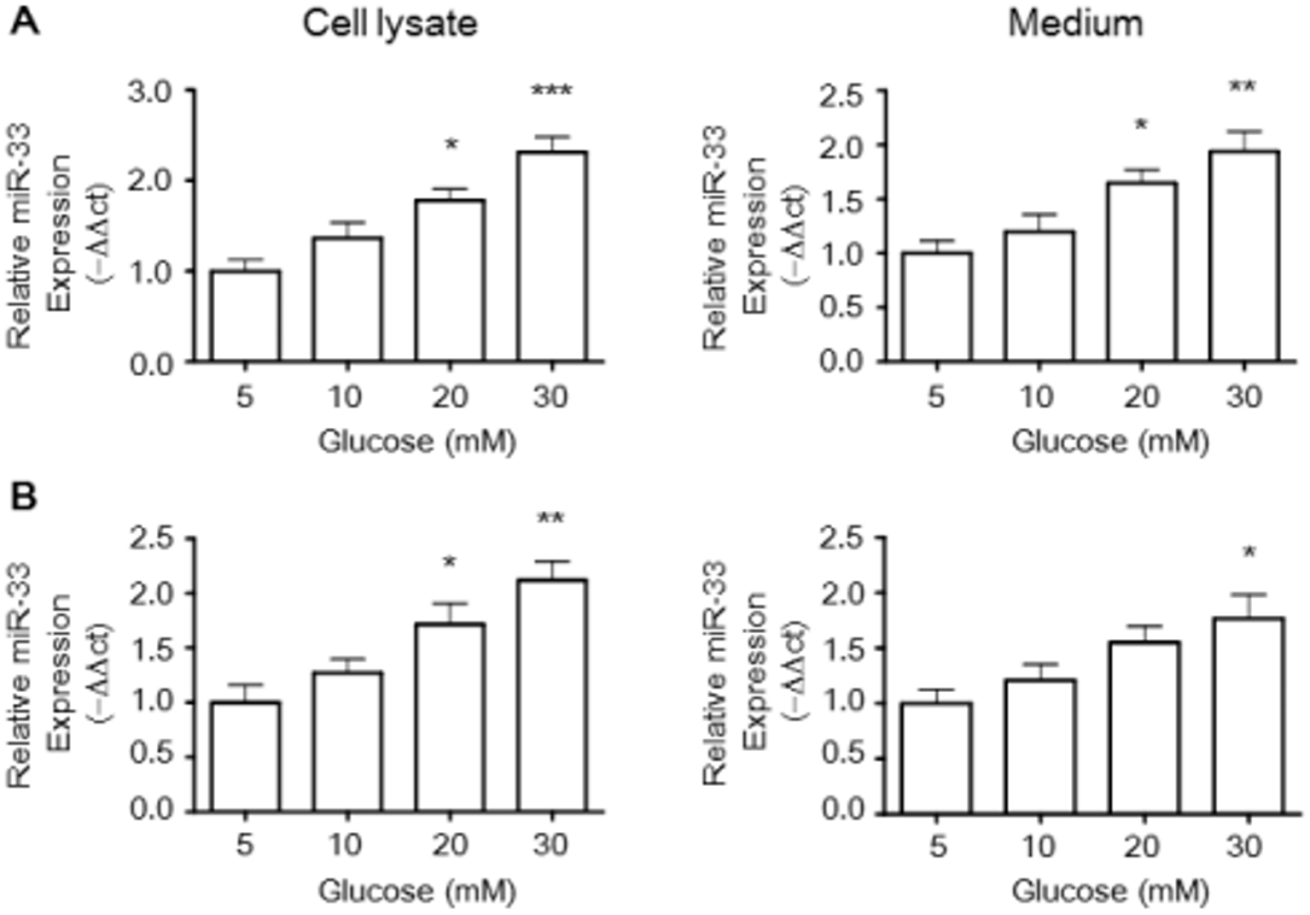
Glucose stimulates miR-33 expression in RAW264.7 macrophages and BMDMs. The effect of glucose on miR-33 levels in the cell lysate and medium was investigated in Raw264.7 murine macrophages (A) and BMDMs (B) by quantitative miRNA real-time PCR. Trizol reagent was used to extract total RNA in cellular lysates and medium from macrophages treated with glucose from 5 to 30 mM. Preparation of microRNA was isolated using the mirVANA microRNA isolation kit and microRNA assays were performed by real-time PCR. The relative miR-33 expression was calculated via the 2^−ΔΔCt^ method using cel-miR-39 as an endogenous control. The histogram shows the relative fold compared with the control group (set as 1). Results are expressed as mean±SEM of at least three independent experiments. *P < 0.05, **P < 0.01, ***P < 0.001 versus control group.

Macrophages were chosen as a cellular model in which to evaluate the regulation of glucose and statins on miR-33 expression. The results of miRNA-specific real-time PCR showed that glucose exposure significantly increased the levels of miR-33 in cell lysates and medium from murine RAW264.7 macrophages (Fig 2A). In addition, Fig 2B showed that glucose dose-dependently increased relative miR-33 expression in comparison with the control group in cell lysates and medium from BMDM cells.

**Fig 3:**
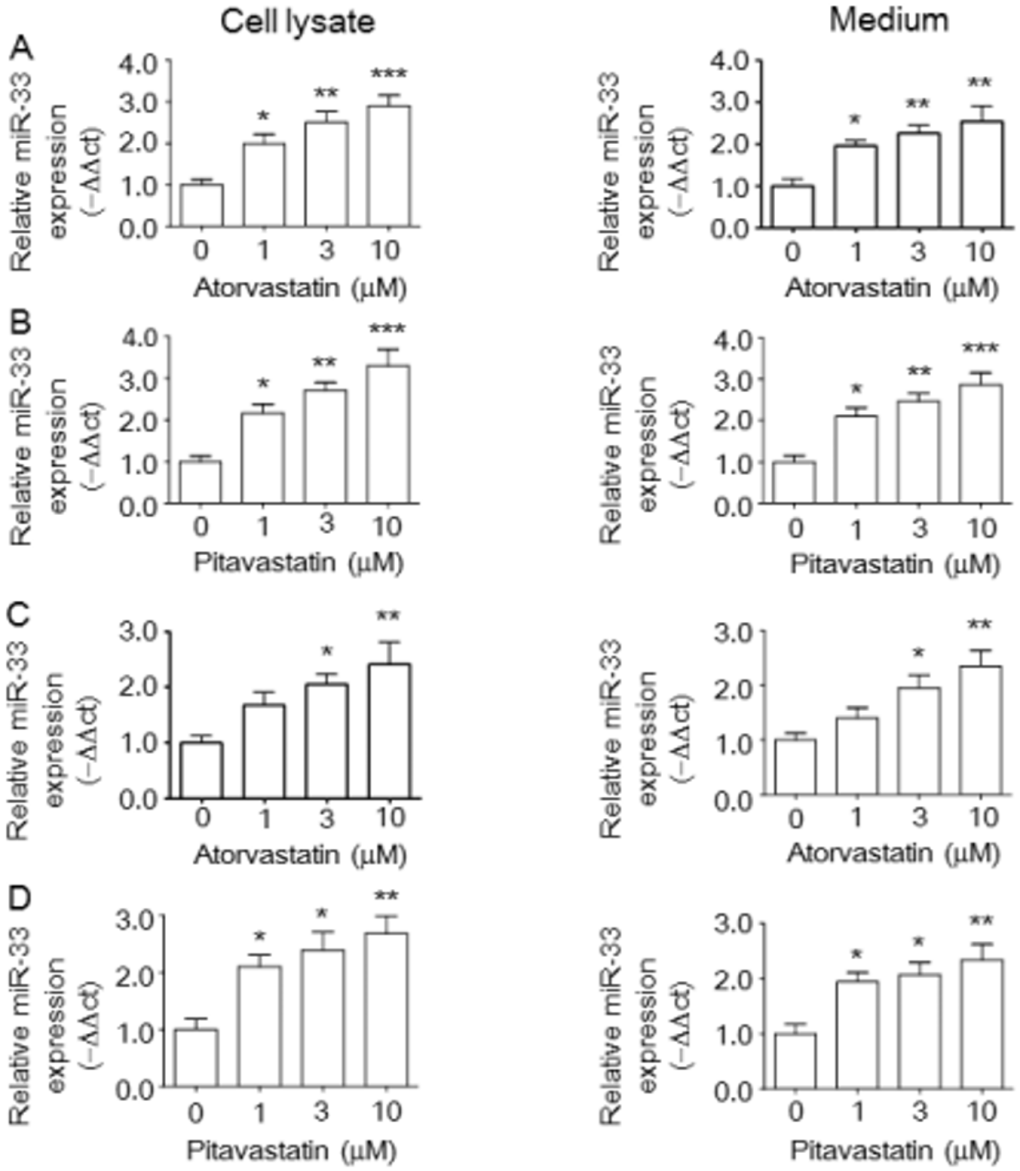
Statins stimulate miR-33 expression in RAW264.7 macrophages and BMDMs. The effect of atorvastatin or pivatostatin on relative miR-33 levels in the cell lysate and medium was investigated in Raw264.7 murine macrophages (A,B) and BMDMs (C,D) by quantitative miRNA real-time PCR as described in the Materials and Methods. The histogram shows the relative fold of miRNA level compared with the control group (set as 1). Results are expressed as mean±SEM of at least three independent experiments. *P < 0.05, **P < 0.01, ***P < 0.001 versus control group.

To assess miR-33 regulation in response to statin treatment, we analyzed the effects of atorvastatin and pitavastatin on miR-33 expression in RAW264.7 macrophages and BMDM cells. Atorvastatin (Fig 3A) and pitavastatin (Fig 3B) dose-dependently up-regulated relative miR-33 expression in cell lysates and medium from RAW264.7 macrophages. Remarkably, increased miR-33 expression was also found in cell lysates and medium from BMDM cells treated with atorvastatin (Fig 3C) and pitavastatin (Fig 3D).

### Statin-mediated miRNA-33-targeted *ABCA1* gene regulation in macrophages

**Fig 4:**
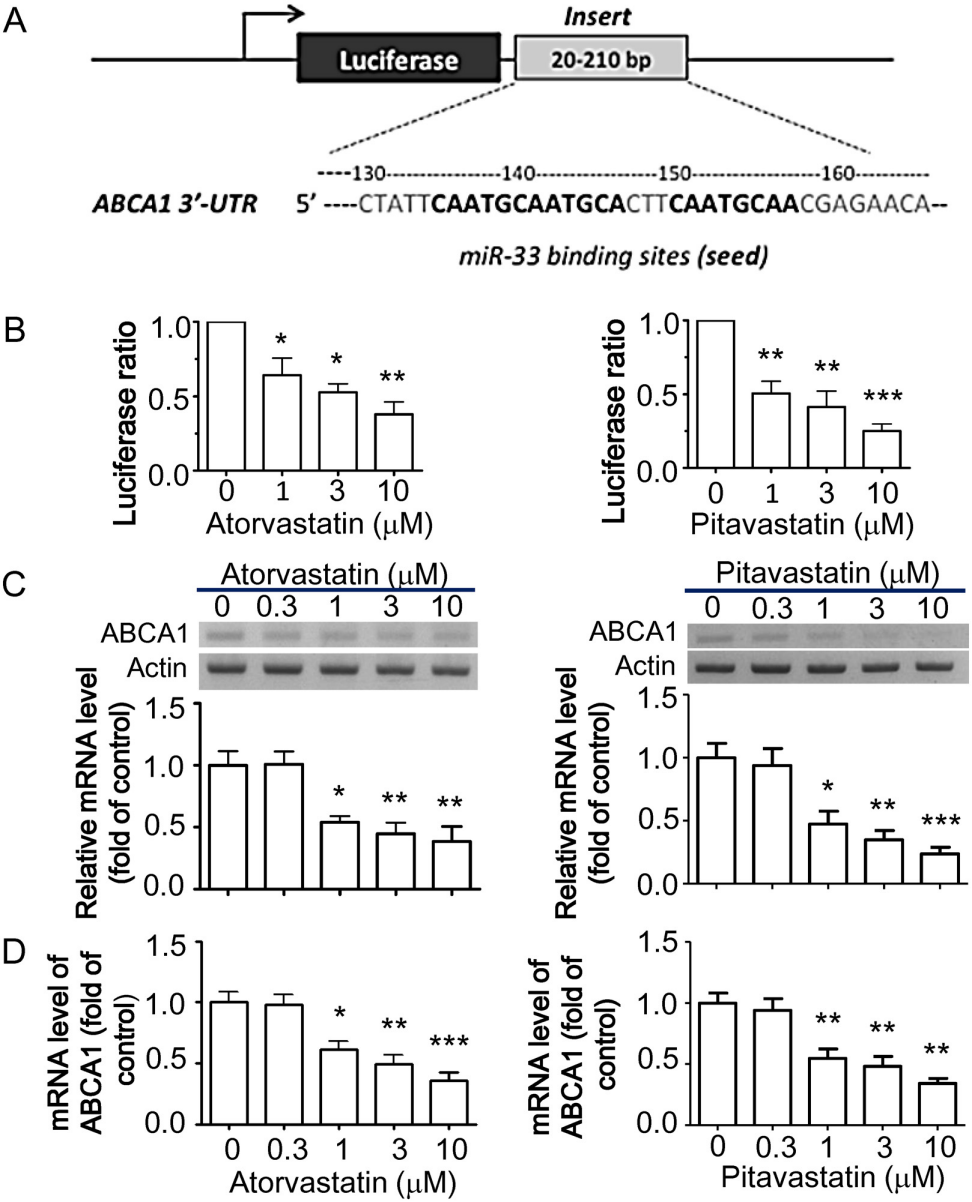
*ABCA1* 3'-UTR activity and relative miR-33 expression after statin treatment in RAW264.7 macrophages and BMDMs. Reporter containing sequence complementary to miR-33a in 3'-UTR of *ABCA1* was constructed in downstream of the luciferase gene of the pGL3-*ABCA1* plasmid (A). Macrophages were transfected with the pGL3-*ABCA1* plasmid and/or statins for 24 h, and then assayed of luciferase activity. A pCMV-β-galactosidase vector encoding β-galactosidase was co-transfected as an internal control. Dose-dependent effects of atorvastatin and pitavastatin on the luciferase activity were analyzed in RAW264.7 macrophages (B). The luciferase activity was normalized to β-galactosidase activity. Data are expressed as ratios relative to the values obtained from the untreated pGL3-*ABCA1* control group defined as 1.0. The mRNA levels of *ABCA1* were determined by semi-quantitative RT-PCR (C) and quantitative real-time PCR (D) as described in the Materials and Methods. The histogram shows the relative fold of *ABCA1* mRNA levels compared with the control group (set as 1). Results are expressed as mean±SEM of at least three independent experiments. *P < 0.05, **P < 0.01, ***P < 0.001 versus control group.

**Fig 5:**
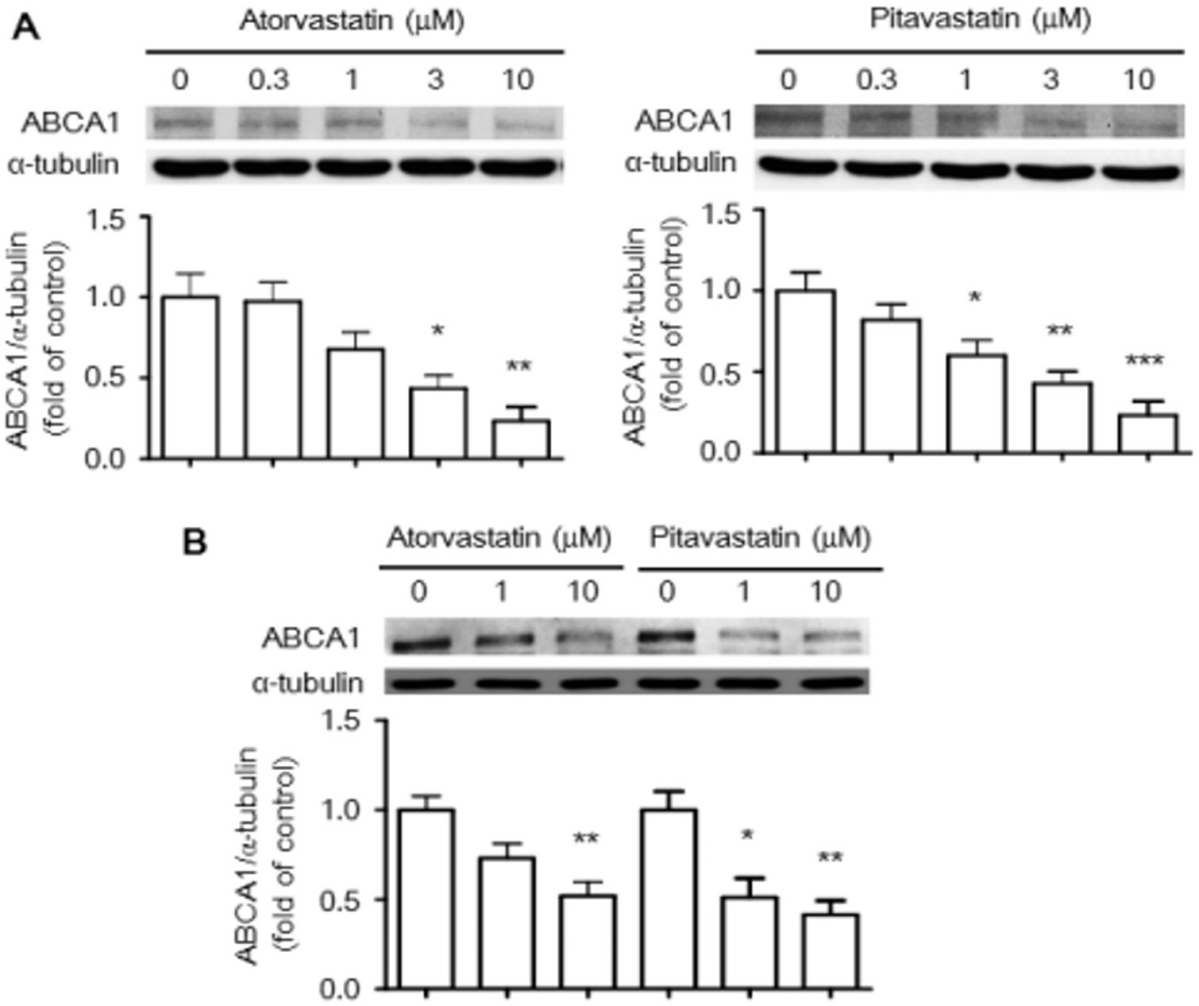
The levels of ABCA1 protein expression in RAW264.7 macrophages and BMDMs treated with statins. Raw264.7 murine macrophages were treated with atorvastatin or pitavastatin at the indicated dosages for 24 h (A). The levels of ABCA1 protein expression were determined by western blot analysis as described in the Materials and Methods. BMDMs were treated with atorvastatin or pitavastatin at 1 and 10 μM for 24 h (B). Results represent mean±SEM of at least three independent experiments. *P < 0.05, **P < 0.01, ***P < 0.001 versus control group.

To confirm *ABCA1* mRNA as a target for statin-induced miR-33, we generated luciferase reporter constructs containing the 3′-UTR of the *ABCA1* mRNA transcript (Fig 4A) and assessed the effects of statins on *ABCA1* gene regulation. Indeed, following transfection of RAW264.7 cells with luciferase reporter constructs, atorvastatin and pitavastatin suppressed relative luciferase activity in a dose-dependent manner (Fig 4B). These results confirm that miR-33 regulates ABCA1 expression by targeting the conserved 3′-UTR of *ABCA1* mRNA. Furthermore, we assessed regulation of mRNA levels of *ABCA1* in macrophages by atorvastatin and pitavastatin using RT-PCR. *ABCA1* mRNA levels were significantly down-regulated by atorvastatin and pitavastatin in RAW264.7 macrophages (Fig 4C). Moreover, the decrease in *ABCA1* mRNA levels was more pronounced in macrophages treated with pitavastatin than in those treated with atorvastatin. Down-regulation of *ABCA1* mRNA levels by statins was also identified by quantitative real-time PCR (Fig 4D). Consistently, ABCA1 protein levels were decreased by atorvastatin and pitavastatin in RAW264.7 macrophages (Fig 5A) and BMDM cells (Fig 5B).

### Down-regulation of cholesterol efflux from macrophages by glucose and statins

**Fig 6:**
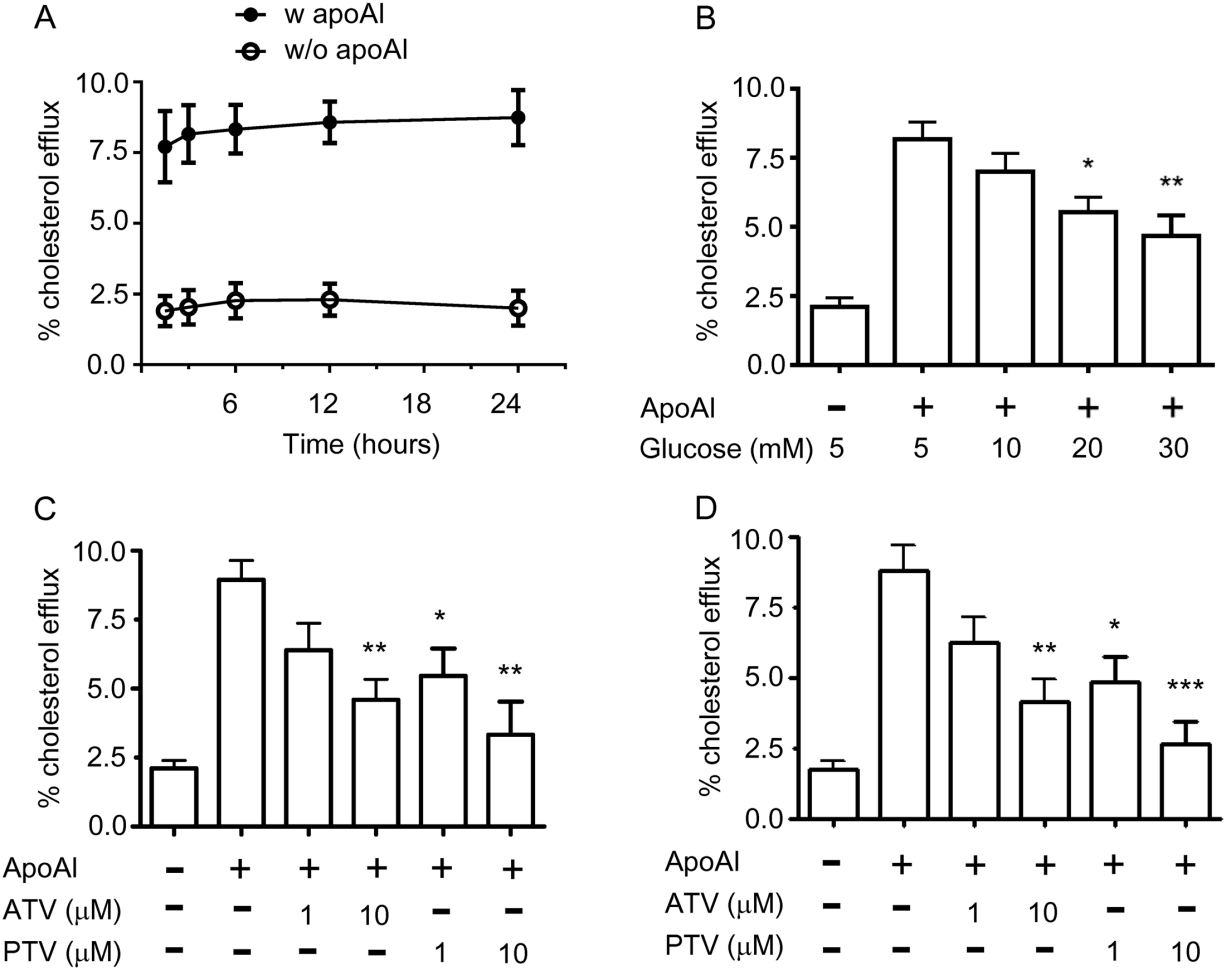
Glucose and statins decrease cholesterol efflux from macrophages. (A) RAW264.7 macrophages were plated with 0.5 μCi/mL [1,2-^3^H]cholesterol, 10 μg/mL acetylated low-density lipoprotein, and 30 μg/mL of cholesterol in DMEM containing 0.2% BSA with 25 μg/mL lipid-free apolipoprotein A1 (apoAI) for the indicated time period (n = 4). Cholesterol efflux was expressed as the percentage of radioactivity in the medium relative to the total radioactivity (medium and cell lysate). (B) RAW264.7 macrophages were treated with different concentrations of glucose as indicated for 24 h in present or absent of apoAI. The effect of glucose on cholesterol efflux was measured as described in (A). RAW264.7 macrophages (C) or BMDMs (D) were treated with atorvastatin (ATV) or pitavastatin (PTV) at 1 and 10 μM in the present or absent of lipid-free human apoA1 acceptor for 24 h and the apoA1-mediated cholesterol efflux was calculated as described in (A). Results represent mean±SEM of at least three independent experiments. *P < 0.05, **P < 0.01, ***P < 0.001 versus control group.

Because ABCA1 can function as mediator for cholesterol efflux [46], we hypothesized that glucose and statins may modulate cholesterol homeostasis through miR33-mediated ABCA1 regulation. To assess the optimal time for the apoAI-mediated cholesterol efflux, RAW264.7 macrophages were incubated with apoAI from 1.5 h to 24 h (Fig 6A). We observed that apoAI started its cholesterol acceptor function from 1.5 h and maintained its cholesterol exporter ability up to 24 h. As shown in Fig 6B, high glucose significantly decreased cholesterol efflux in a concentration-dependent manner compared to control cells cultured with 5mM glucose. Furthermore, atorvastatin and pitavastatin also reduced cholesterol efflux from RAW264.7 macrophages (Fig 6C) and BMDM cells (Fig 6D). Taken together, these findings suggest that alterations in miR-33 levels and ABCA1 expression by glucose and statins decrease cholesterol efflux from macrophages.

## Discussion

Aberrant cholesterol homeostasis is linked to a number of diseases, including MetS, type 2 diabetes, and cardiovascular disease. We have previously discovered a post-translational regulatory mechanism underlying hyperglycemia [21]. Subjects with MetS generally have a combination of hyperglycemia and atherogenic lipid profiles, including low HDL-C and high non-HDL-C levels. Many clinical trials have shown that statins effectively reduce LDL-C levels and/or increase HDL-C levels, although Sasaki et al. reported that prolonged use of atorvastatin and pitavastatin reduced HDL-C levels [30]. Statin treatment is commonly used to lower cholesterol levels in populations with hypercholesterolemia [26,27], yet pleiotropic effects of statins have been identified recently [22–25].

We expand the knowledge of how hyperglycemia and statins contribute to cholesterol homeostasis and their underlying mechanisms in the present study. Cholesterol efflux from macrophages is a key mechanism to prevent the development of atherosclerosis [47,48], however, little is known about the mechanism by which statin therapy leads to cholesterol transportation in macrophages. Moreover, limited datum is available for the effects of statins on miRNA levels involved in the therapy of MetS. Emerging evidence demonstrate that miRNAs have gained considerable attention as therapeutic targets [3,5,8,9]. We thus explore the possibility by which statins regulate miRNA expression in the MetS patients as well as the statin-mediated disturbance of cholesterol homeostasis in macrophages.

Elevation of miR-33, miR-103, and miR-155 levels has been found to involve the dysfunction of cholesterol transportation, insulin resistance, and glucose homeostasis, respectively [10–12]. However, the present study shows for the first time that increased plasma miR-33 expression is positively correlated with levels of fasting blood glucose, HbA1c, TC, LDL-C, and TG, but negatively correlated with HDL-C levels in human subjects. Moreover, our population-based study showed that miR-33 expression was increased in subjects after treatment with atorvastatin and pitavastatin. To explore whether statins involve the miR-33-mediated *ABCA1* gene regulation, we constructed a luciferase reporter containing the 3′-UTR of the *ABCA1* mRNA transcript. Consistent with the prediction, we found that statins control the *ABCA1* gene expression via harboring the *ABCA1* 3′UTR miR-33 binding sites. These findings suggest that miR-33 levels interconnect metabolic regulatory network governing cholesterol homeostasis via a post-transcriptional regulation of the *ABCA1* gene. In accord with the data that miR-33 and ABCA1 may be functionally linked, we showed that ABCA1 and cholesterol efflux are co-regulated by glucose and statins in macrophages, suggesting that hyperglycemia and statins seem to share a common regulatory mechanism.

Previous reports show that miR-33 is encoded by an intron of *sterol regulatory element-binding protein 2* (S*REBP2*) gene and has been shown to play a crucial role in interfering cholesterol homeostasis [10,49]. Two isoforms of miR-33 exist in humans: miR-33a, which is located in intron 15 of the *SREBP2* gene on chromosome 22, and miR-33b, which is present in intron 17 of the *SREBP1* gene on chromosome 17. In mice, there is only one miR-33a isoform, which is located within intron 15 of the mouse *SREBP2* gene [50]. Accordingly, the mature miR-33a/b appears to be co-expressed with the *SREBP* host genes in a number of human and mouse tissues. Wong et al have shown that SREBP2 acts as a positive regulator of *ABCA1* gene expression by oxysterol ligands [51]. Moreover, miR-33 regulates post-transcriptional expression of AMPK-activated protein kinase (AMPK), insulin receptor substrate 2 (IRS2), and NAD+-dependent deacetylase 6 (SIRT6), thus controlling glucose and triglyceride metabolism [52,53]. However, we expand a link between miR-33-mediatead ABCA1 regulation and cholesterol homeostasis by high glucose and statins in this study. Under conditions of hyperglycemia and statin treatment, microRNA-33 levels are elevated, and ABCA1 expression is decreased, which then results in a reduction in cholesterol efflux from macrophages.

Such studies should allow the broader impact of miR-33 antisense targeting to be addressed. The potential role of antisense oligonucleotide targeting of miR-33 could indeed be a clinically useful approach in improving cholesterol homeostasis, raising HDL and ameliorating MetS. It has been reported that ABCA1 expression and cholesterol efflux are found to be impaired in patients with type 2 diabetes [54]. With the expansion of the role of miR-33 in the regulation of cholesterol homeostasis, therapeutic benefits of miR-33 antisense targeting may encompass not only ameliorated MetS, but also its effects on the broader array of disease associated with metabolic syndrome, including insulin resistance, hepatosteatosis, diabetes, and atherosclerosis.

Recently, simvastatin treatment was found to reduce expression of receptor interacting protein 140 (RIP140) by increasing miR-33 expression through recognition of a highly conserved sequence in the 3′-UTR of the *RIP140* mRNA transcript in macrophages [55]. Nevertheless, no comprehensive mechanism has been proposed to explain the relationship between statin therapy and changes in cholesterol homeostasis in macrophages. In the present study, we showed that the conserved miR-33-responsive element sequence in the 3′-UTR of the *ABCA1* gene was activated by atorvastatin and pitavastatin administration, which may interfere with the beneficial effects of statins as therapeutic interventions in patients with diabetic dyslipidemia and as preventive measures against CVD.ABCA1 is an exporter of cholesterol from macrophages, an essential determinant of plasma HDL levels, and a potent cardioprotective factor [14]. Therefore, ABCA1 has become a new therapeutic target for treatments aimed at removing cholesterol from arterial macrophages, as well as preventing CVD and diabetes [16,18,54]. In this study, we found that statins altered cholesterol transportation in macrophages through miRNA-33a-mediated *ABCA1* gene regulation. Ultimately, decreased ABCA1 expression results in reduced cholesterol efflux from macrophages, which may lead to atherosclerotic progression. Actually, no drug is entirely free of adverse effects. Growing concern about the risk inherent in the high frequency of statin prescriptions should thus be considered. Nevertheless, another possible explanation for our finding is that the effect of statin on stimulation of miR-33 and decrease in ABCA1 expression is a compensatory mechanism to preserve intracellular cholesterol stores. Further studies are required to elucidate the physiological meaning of the opposing regulation of statins on cholesterol efflux from macrophages.

Taken together, we elucidate that miR-33 acts in a concerted manner with its target *ABCA1* gene products to regulate cholesterol homeostasis in macrophages. Our data provide evidence that high glucose and statins decrease cholesterol efflux from macrophages through miR-33-mediated down-regulation of ABCA1 expression. It would thus represent the first example that miRNA–host gene cooperativity regulated by statins and the MetS metabolite glucose. The down-regulation of ABCA1 expression associated with statin use may facilitate the development of novel therapeutics, such as a new generation of statins with fewer adverse effects.
